# Discretionary Food Consumption Patterns of Polish Schoolchildren in Relation to Anthropometric, Socio-Demographic, and Lifestyle Factors: Report from the Junior-Edu-Żywienie (JEŻ) Project

**DOI:** 10.3390/nu17081378

**Published:** 2025-04-19

**Authors:** Małgorzata Ewa Drywień, Jadwiga Hamulka, Ewa Czarniecka-Skubina, Jerzy Gębski, Małgorzata Kostecka, Krystyna Gutkowska

**Affiliations:** 1 Department of Human Nutrition, Institute of Human Nutrition Sciences, Warsaw University of Life Sciences SGGW (SGGW-WULS), 166 Nowoursynowska Street, 02-787 Warsaw, Poland; malgorzata_drywien@sggw.edu.pl (M.E.D.); jadwiga_hamulka@sggw.edu.pl (J.H.); 2 Department of Food Gastronomy and Food Hygiene, Institute of Human Nutrition Sciences, Warsaw University of Life Sciences SGGW (SGGW-WULS), 166 Nowoursynowska Street, 02-787 Warsaw, Poland; ewa_czarniecka-skubina@sggw.edu.pl; 3 Department of Food Market and Consumer Research, Institute of Human Nutrition Sciences, Warsaw University of Life Sciences SGGW (SGGW-WULS), 166 Nowoursynowska Street, 02-787 Warsaw, Poland; jerzy_gebski@sggw.edu.pl; 4 Department of Chemistry, Food Science and Biotechnology, University of Life Science in Lublin, 13 Akademicka Street, 20-950 Lublin, Poland; malgorzata.kostecka@up.lublin.pl

**Keywords:** adolescents, discretionary food, dietary patterns, anthropometric, lifestyle, nutrition knowledge

## Abstract

**Background:** Discretionary foods are not necessary for a healthy diet and are too high in saturated fat and/or added sugars, added salt, or alcohol and are low in fiber. The aim of this study was to identify patterns of discretionary food (DF) consumption in Polish adolescents in relation to anthropometric, socio-demographic, and lifestyle factors. It is important to learn about discretionary food consumption habits to identify health risks and plan strategies to reduce DF consumption. **Methods**: The cross-sectional study was conducted among 2849 primary school students aged 10–12 from Poland. Socio-demographic data, eating habits, lifestyle factors, and nutritional knowledge of children were assessed using questionnaires: KomPAN^®^ and SF-FFQ4PolishChildren^®^. Body composition testing was performed by bioelectrical impedance analysis (BIA) using a TANITA MC-780 S MA multi-frequency segmented analyzer. **Results**: Four dietary patterns were identified with different frequencies of discretionary food consumption: LowDF, MediumDF, HighDF, and HighSweet pattern. Children from the LowDF and MediumDF patterns had higher BMI and body fat content than children from the HighDF and HighSweets patterns (*p* < 0.0001). The majority of children (68.4%) were of normal weight, 22.3% were overweight or obese, and 9.4% were underweight. Children in the LowDF and MediumDF patterns had higher body fat (24% and 23.5%, respectively) than children in the HighDF and HighSweetDF patterns (22.4% and 22.4%, respectively). Most of the children showed adherence to the MediumDF pattern, and they were mainly boys (38 vs. 32.5%). Girls predominate in LowDF, HighDF, and HighSweets patterns (33.5, 18.4, and 15.5%, respectively). Children in the LowDF and MediumDF patterns tended to be more physically active in their leisure time (OR = 1.758 (CI:1.32 2.34) *p* < 0.0001; OR = 1.354 (CI:1.04; 1.76) *p* = 0.0227) and the opposite relationship was observed in the HighDF pattern (OR = 0.495 (CI:0.38; 0.65) *p* < 0.0001). Children in the HighSweets pattern had low adherence to high physical activity (OR = 0.666 (CI:0.48; 0.92) *p* = 0.0132), but high adherence to moderate (OR = 1.29 (CI:1.01; 1.65) *p* = 0.0443) and high (OR = 1.54 (CI:1.04; 2.38) *p* = 0.0428) nutrition knowledge. **Conclusions**: Consumption of discretionary foods by Polish adolescents is related to body composition, socio-demographic, and lifestyle factors. Given the global emphasis on improving the daily diet, it seems necessary to implement intervention programs in Poland that would, among other things, clarify recommendations for the consumption of discretionary foods, following the example of other countries that have already achieved results in this regard. From a public health perspective, interventions to increase nutritional knowledge and improve lifestyles should be implemented with both adolescents and their parents in coordination with the school.

## 1. Introduction

Discretionary foods (DF), defined by the Australian Dietary Guidelines (ADGs) as foods high in added sugar, salt, saturated fat, and alcohol, include cakes, biscuits, confectionery, cookies, ice cream, butter, cream, spreads high in saturated fat and/or added sugar, crisps and other salty snacks, and sugar-sweetened beverages, including soda, soft drinks, sports drinks, and energy drinks. Simply put, it is an energy-rich, nutrient-poor food that is popular because it tastes good and gives a sense of pleasure [1]. The literature shows that DF is consumed from an early childhood and, according to [2], contributes 16% of energy, 21.5% of lipids, 34% of sodium, and about 75% of free sugars to the daily ration. Other Australian research reported that children and adolescents (2–18 years) receive almost 40% of their daily energy intake from discretionary foods [3]. The Dutch National Food Consumption Survey reports that 18–21% of energy consumption (E%) in children and adolescents (7–18 years) comes from free sugars, of which over 80% comes from sweets, candy, cakes, and sugar-sweetened beverages [4]. While Danish dietary survey data conclude that Danish preschool children consume too much discretionary foods, on average, they have an intake of 125 g/week of candy and chocolate, 385 g/week of cakes, ice creams, and energy-dense snacks [5].

Discretionary food is not a nutritionally necessary food and also contributes to the formation of inappropriate eating habits and increased health outcomes in children, such as weight gain and its consequences, tooth decay and cardiovascular risk factors [6,7,8,9,10,11,12]. Studies in children have shown that discretionary choices (energy-dense, nutrient-poor “extras”) can replace staple foods such as fruits, vegetables, dairy products, lean meats, and whole grains [13] as evidenced by the contents of children’s lunchboxes [14,15]. Limiting the current consumption of discretionary foods will reduce the risk of nutrient deficiencies, obesity, and associated chronic diseases [1]. To date, only Australia and New Zealand (Australian Government) [1], and the USA [16] and Scotland [17] have defined discretionary foods and introduced recommendations for their consumption, and Denmark has recently joined them [18]. Denmark has chosen the new maximum recommended intake of discretionary foods and defined it as 4–6% of total energy consumption, where discretionary foods and drinks include chocolate, candy, salty snacks, sugar-sweetened beverages, cakes, and desserts [19]. These new guidelines have been communicated as limits of weekly servings and small servings for children and are now part of the Danish official dietary guidelines [20]. It is possible to implement discretionary food consumption reduction scenarios with positive results (reducing energy intake by up to a third), with the greatest energy reductions observed when all discretionary foods (excluding beverages) are removed, large portions are removed, and these foods are not consumed as part of main meals. Further research is needed to adapt these reduction scenarios to children’s eating environments [21]. The variety of discretionary foods/beverages is related to total consumption, so intervention approaches aimed at reducing variety would be a novel way to counteract overconsumption of discretionary foods/beverages, as an alternative to focusing on portion size or frequency of food consumption. On the other hand, manipulating the variety of discretionary food consumption has been studied as a strategy to increase healthy food consumption and has been positively associated with higher vegetable consumption [22]. Recently, it has been suggested that reducing the production and consumption of discretionary foods could be a key step in creating a more sustainable food system [23,24].

A systematic review by Maneschy [25] presented an analysis of existing research on the association between eating behavior and food and beverage intake in children and adolescents, with attention to dietary approaches and the intake of discretionary food. Concerningly, an unhealthy diet and consumption of discretionary foods is known to commonly cluster with high screen time in children and adolescents and may have adverse impacts on health outcomes [26]. A cross-sectional study conducted by Fletcher et al. [27] on adolescents showed that watching television (≥2 h/d) was positively associated with consuming discretionary snacks at least once daily, whereas computer use (≥2 h/d) was inversely associated with daily fruit and vegetable intake and positively associated with weekly fast-food consumption. Total screen (≥2 h/d) and sitting (h/d) times were also positively associated with daily discretionary snack consumption and weekly consumption of SSB and fast foods. Studies have shown that prolonged screen time may be linked to increased childhood obesity, poorer sleep quality, and mental health problems, such as increased stress and anxiety [28,29]. It seems that by focusing on physical activity and sedentary behavior, researchers may inadvertently be overlooking the intricate interplay and synergistic effects added by diet and sleep, risking creating an oversimplified understanding of the clustering of health behaviors [26]. The functioning of an organism and its physical quality are positively correlated with mental health and its dimensions, such as cognitive functions, emotions, and personality [30]. Shaping behavior in the context of lifestyle and nutritional behavior during childhood and adolescence may have an impact on behavior in adulthood.

There is limited evidence on the effectiveness of dietary intervention strategies to reduce discretionary choices. Improving the understanding of dietary intervention strategies that are potentially relevant for reducing discretionary food consumption will inform the design of the next generation of interventions needed to prevent overweight and obesity and/or reduce non-communicable chronic disease risk factors [14]. Therefore, the aim of this study was to identify patterns of discretionary food consumption in Polish adolescents in relation to body composition and socio-demographic and lifestyle factors in order to identify health risks and plan strategies to reduce the consumption of discretionary food in adolescents.

## 2. Materials and Methods

### 2.1. Ethics Approval

The study was approved by the Ethics Committee of the Faculty of Human Nutrition and Consumer Sciences at the Warsaw University of Life Sciences (Resolution No. 18/2022). The guidelines of the Declaration of Helsinki were followed during the study. Parents gave informed consent for their children’s participation in the study through the signing of a consent form. Students who refused to participate in the study were excluded from the procedures. The children (pupils) who did not take part in the research were under the care of the teachers and took part in other school activities.

### 2.2. Study Design and Participants

This cross-sectional survey was conducted in June 2022–November 2023 as part of the Junior-Edu-Żywienie (JEŻ) project among a representative sample of students aged 7–12 from schools in all 16 Polish provinces in five different macro-regions: Central (Masovian Voivodeship, Łódź Voivodeship); Northeast (Warmian-Masurian Voivodeship, Podlaskie Voivodeship, Lublin Voivodeship); Northwest (Pomeranian Voivodeship, West Pomeranian Voivodeship, Kuyavian-Pomeranian Voivodeship and Greater Poland Voivodeship); Southwest (Lubusz, Lower Silesia, Opole and Silesia Voivodships); and Southeast (Swiętokrzyskie, Lesser Poland and Podkarpackie Voivodships). Additionally, for the purposes of this study, the regions have been grouped geographically into the following: Central, Eastern, and Western. The study was conducted according to the procedures described by Hamulka et al. [31] in detail.

Of the 12,695 children between the ages of 10 and 12 who were recruited for the project, 2849 had their body composition measured if their parents, and the children themselves, consented. Children who were afraid of the procedure and withdrew immediately before the anthropometrics measurement (body composition) or were absent from school on the day of the measurement were not measured. The questionnaire survey collected data on the gender, age of the adolescents, and type of residence: rural or urban (villages, towns with up to 100,000 inhabitants, or towns with more than 100,000 inhabitants).

### 2.3. Data Collection

#### 2.3.1. Dietary Assessment

The children’s dietary habits, lifestyle factors, and nutritional knowledge, as well as their socio-demographic data, were assessed using a questionnaire developed for the project. The questionnaire was based on a validated tool for Polish adolescents, the SF-FFQ4PolishChildren^®^ [32].

The questionnaires were developed for the project, but were based on validated and published questionnaires. The first part of the questionnaire assessed students’ knowledge of current dietary guidelines and physical activity. In the second part, information on dietary habits was collected, mainly the frequency of consumption of 12 food groups in the 12 months prior to the questionnaire. In the last part, the students indicated their sex, date of birth, and grade in school. Lifestyle factors assessed in children included physical activity, time spent in front of screens, and sleep time. More details on the diet assessment questionnaires were previously described in the study protocol [31].

Five discretionary food groups were selected from 12 food groups: fast food (e.g., French fries, pizza, and hamburgers), sugar-sweetened beverages (e.g., cola, tea, and water with syrup), energy drinks, sweets or confectionery (e.g., cookies, candy, cakes, chocolate bars, and chocolate), salty snacks (e.g., chips and coated peanuts). Finally, energy drinks were excluded from the assessment because their frequency of consumption was low and did not statistically differentiate the identified consumption patterns. The following frequency of consumption was used: 1-never or almost never, 2-less than once a week, 3-once a week, 4-two-four times a week, 5-five-six times a week, daily, and 6-several times a day.

The assessment of general nutrition knowledge was based on 20 closed questions. Five of these questions were used from the validated questionnaire dedicated to Polish youth, SF-FFQ4PolishChildren^®^ [33]. The other 15 questions referred to the current dietary guidelines for Polish children and adolescents [34]. A correct answer was worth 1 point, while other answers were worth 0 points. Scores were summed for each respondent (ranging from 0 to 20 points). Based on the tertile distribution, respondents were divided into three categories of nutrition knowledge: low (0–7 points), medium (8–14 points), and high (15–20 points).

The paper-and-pencil questionnaire was self-administered by the schoolchildren in classrooms under the supervision of researchers and teachers. The researchers provided necessary supporting information as needed and avoided formulating suggestive responses. Each questionnaire was checked for completeness of all responses and then coded for anonymization. The questionnaire took approximately 30–45 min to complete.

#### 2.3.2. Body Composition Assessment

Body composition (BC) measurements were performed in the morning between 8:00 and 12:00 using the same type of professional equipment in all schools. Measurements were not performed on adolescents with arm or leg deformities that prevented proper contact with the electrodes. Before measuring body composition, height was measured in centimeters to the nearest 0.5 cm using a portable stadiometer (TANITA Corporation. Tokyo, Japan). The person was measured from the base of the foot to the top of the head (top), without shoes, in an upright position, with feet close together and heels together, facing away from the stadiometer, with arms positioned along the body and head aligned in the Frankfurt plane.

The body composition test was performed using the bioelectrical impedance (BIA) method, which involves the study of the flow of a very low current through tissues that is harmless to health. The measurement was performed with the TANITA MC-780 S MA (TANITA Corporation, Tokyo, Japan)—a multi-frequency segmented body composition analyzer with an 8-point electrode system. Subjects were measured in a standing position after removal of metal items (jewelry and watches), at least 2 h after a meal and at least 12 h after intense physical activity. Before the measurement, the subjects had to empty their urinary bladder. The detailed procedures were previously described in paper [31].

The results obtained regarding skeletal muscle mass (MM) and fat-free mass (FFM) were compared to available reference values [35], and fat mass (FM) was compared to reference values provided in the TANITA manual based on McCarty et al. [36]. Body mass status was classified based on BMI values for underweight, normal weight, overweight, and obesity using the TANITA GMON MDD PROFESSIONAL Health Monitor (Medizin & Service GmbH; Boettcherstr. 1009117 Chemnitz, Germany) [37].

### 2.4. Statistical Analysis

As a preliminary analysis of the results, an analysis of the distributions of the variables was carried out. The characteristics of the sample are presented according to the gender of the respondents. The normality of the distribution of continuous variables was assessed with the Kolmogorov–Smirnov test. The clustering was based on the k-means method, using the centroids of the patterns derived from the hierarchical method. This resulted in 4 well-separated clusters (patterns). The number of patterns was selected based on the dendrogram and statistics: CCC (Cubic Patterning Criteria) and Pseudo F. In addition, the correct level of pattern differentiation was confirmed by the ANOVA analysis of variance from the post-hoc Waller–Duncan k-ratio *t*-test.

Profiling of the selected patterns was carried out using variables describing the socio-demographic characteristics of the respondents, eating habits, etc. For categorical variables, the test of independence Chi2 was used, and for quantitative variables, the post-hoc Waller–Duncan K-ratio *t*-test was used. All statistical analyses were performed using the SAS 9.4 software package at a significance level of *p* < 0.05.

Frequency of fast food consumption is a variable that was included in the clustering process, but its level is the lowest of the variables used. Associations between dietary patterns and selected characteristics of the study adolescents was calculated as odds ratios.

## 3. Results

### 3.1. Characteristics of the Study Population

The majority of children surveyed were 10 years old from medium-sized cities or eastern macro-regions (Table 1). Almost half of the children reported high levels of leisure-time physical activity, and the majority of these were boys. More than one-third of respondents reported 2–4 h of screen time, and more than half reported an average of 6–8 h of sleep. Most of the children surveyed had a BMI indicating a normal body weight and reported a moderate knowledge of current nutritional recommendations.

#### 3.1.1. Dietary Patterns Characteristics

Cluster/Pattern 1 (LowDF) was characterized by a low frequency of consumption of all the discretionary products analyzed (Table 2). The LowDF Pattern was characterized by the highest percentage of girls, children aged 10 years, living in rural areas or large cities, and coming from the central macro-region. It had the highest proportion of children reporting average physical activity, less than 2 h of screen time per day, more than 8 h of sleep, and good nutrition knowledge (Table 3).

In Cluster/Pattern 2 (MediumDF), the average frequency of discretionary food consumption was similar to the average of the sample as a whole (Table 2). The MediumDF Pattern had the largest proportion of boys, children aged 11 years, from mid-sized cities, and eastern macro-regions. Representatives of this cluster reported low levels of physical activity, more than 6 h of screen time, less than 6 h of sleep, and average nutrition knowledge (Table 3).

Cluster/Pattern 3 (HighDF) was characterized by the most frequent consumption of all types of DF, especially of products with added sugar, and by the average consumption of fast food (Table 2). The HighDF Pattern was characterized by mostly boys, children aged 11–12 years, from medium-sized cities, and western macro-regions. Children in this cluster reported average leisure-time physical activity, 4–6 h of screen time, 6–8 h of sleep, and poor nutrition knowledge (Table 3).

Cluster/Pattern 4 (HighSweets) was characterized by the highest frequency of the consumption of sweets or confectionery and by the average of the other types of DF (Table 2). The HighSweets Pattern had the highest proportion of girls, the youngest children (10 years old), those living in rural areas, and those from the central macro-region. Children reported low levels of physical activity, more than 6 h of screen time, less than 6 h of sleep, and good nutrition knowledge (Table 3).

#### 3.1.2. DF Consumption Patterns vs. Anthropometrics

The LowDF and MediumDF patterns were represented by overweight and obese children with above average BMI, percentage fat mass, skeletal muscle mass, and lean body mass. (Table 4). The HighDF pattern was characterized by children with lower (near average) levels of all measured body components and lower BMI; most children were underweight. The HighSweets pattern was characterized by children with BMI and body fat percentages similar to the previous cluster, while the other body composition parameters had the lowest values.

### 3.2. Predictors of the Dietary Patterns

The results of logistic regression analysis are presented in Table 5.

Children from medium-sized cities, from western macro-regions, or who reported a screen time of more than 2 h per day were less likely to belong to the LowDF pattern. In contrast, children who reported moderate to high levels of leisure-time physical activity, who slept more than 6 h, who reported good nutritional knowledge, or who were obese were more likely to be in the LowDF pattern.

Children aged 11–12 years, from medium-sized cities, from eastern macro-regions, who reported medium to high leisure-time physical activity, a screen time over 2 h, and children with obesity were more likely to adhere to the MediumDF pattern.

The HighDF pattern was characterized by a lower likelihood of adherence for children from medium and large cities, children who slept more than 6 h, children with medium to good knowledge of dietary recommendations, or children who were overweight or obese. In contrast, children who reported more than 2 h of screen time were more likely to comply.

Children aged 11–12 years, those from eastern macro-regions, those who reported high leisure-time physical activity, those with normal or excessive body mass, and those who were obese were more likely to belong to the HighSweets pattern, while children who reported moderate to good knowledge of dietary recommendations were more likely to belong to this pattern.

## 4. Discussion

As a result of research conducted among Polish children aged 10–12 years, four patterns of discretionary food consumption were identified that were differentially associated with body composition, socio-demographic factors, and lifestyle. The three patterns differed in the frequency of consumption of the listed products, with the frequency of consumption increasing evenly from LowDF to HighDF and being dominated by products containing sugar. The fourth pattern differed significantly in the structure of the frequency of consumption of products, with a significant proportion consisting of the sweets or confectionery group. They are a group of products that are high on the list of major sources of energy, saturated fat, sodium, and/or added sugars and are ubiquitous in terms of consumption by people of all ages. Therefore, it is emphasized that reducing their consumption may have the greatest impact on improving diets [3]. The trend of increasing consumption of sugary products among school children is characteristic of different countries [38,39,40,41,42]. It is a global problem, and attempts to explore it in more depth are increasingly appearing in the scientific literature. In addition to its association with overweight and obesity, dental disease, and others, there is information linking, for example, high consumption of sugar-sweetened beverages with negative effects on stress, depressive symptoms, and suicidal ideation [43,44,45].

Interesting results were obtained for the anthropometric parameters of children from the LowDF and MediumDF patterns, who had higher BMI and body fat content than children from the HighDF and HighSweets patterns. Obese children were also less likely to follow the HighDF and HighSweets patterns, as observed in previous studies [46,47,48], so other factors are important here. This is in contrast to other findings that did not find such associations [41,42]. Underweight adolescents were more likely to adhere to the HighSweets pattern than the others. Given the results of our study, it is possible that children with a tendency to gain weight may have had their DF consumption restricted by their parents, or, perhaps, they were taking care of it themselves, as they had a higher concordance with good nutritional knowledge and moderate to high leisure-time physical activity in the LowDF pattern. It is possible that parents of obese children view limiting their child’s intake of high-calorie and unhealthy foods as critical to their child’s current and long-term health and well-being. To this end, they may use directive imperatives with young children, and this approach may be adaptive and appropriate in adolescence [49].

Other deeper causes may be behavioral mechanisms and psychological aspects that we did not assess, but which may be relevant to both underweight and overweight people. Emotional eaters tend to make unhealthy, taste-driven food choices under the influence of positive or negative emotions [50].

For adolescents belonging to HighDF and SweetDF patterns with low BMI, emotions may have influenced hedonistic eating (i.e., taste-oriented consumption of high-calorie, low-nutrient foods high in sugar, salt, and fat for pleasure without hunger) or homeostatic eating (i.e., hunger-oriented food consumption to regulate energy balance) in different contexts [51,52,53]. Sensory experiences can be made visible in mental states and emotions through repeated associations between taste and emotion (e.g., associations between sweet taste and happiness) early in life [54,55]. Another marker may be “cheat meals”, described in the literature as eating episodes that temporarily deviate from established dietary practices (i.e., restrictive and/or restrained) in order to temporarily consume forbidden foods, only to return to previous dietary practices (i.e., a “cheat” deviation from regular rigid dietary practices) [56]. This is a muscularity-oriented diet that alters the physique and manipulates the body through the consumption of high-calorie meals [56]. The results of a Canadian study [57] confirm the use of cheat meals in more than half of the adolescents and young adults who participated in the study. Participation in cheat meals was associated with higher rates of eating disorder behaviors and psychopathology.

For adolescents with high BMI, one explanation for adherence to LowDF and MediumDF patterns may be the likelihood of avoidant/restrictive food intake disorder (ARFID), which is associated with obesity and an increased risk of comorbid psychiatric disorders (anxiety, depression, and neurodevelopmental and disruptive behaviors, even including suicidal tendencies) [58]. Childhood obesity is closely linked to depression, which can be exacerbated by the stigma, teasing, and bullying often experienced by overweight adolescents [59]. Those with overweight/obesity were more likely to meet the ARFID diagnostic criteria for psychological distress. In addition, more than half of participants with overweight/obesity (overweight/obesity) met the criteria for comorbid anxiety disorders and almost 20% met the criteria for depression. Both ARFID and obesity independently increase the risk of psychiatric sequelae, and further research is needed to better understand the interaction of these two factors in adolescents with ARFID and obesity [60].

It has been shown that children with higher body weights have greater food sensitivities, and as they become more food-focused, their parents may need to use more directive imperatives to achieve optimal long-term outcomes for the child’s health and wellbeing [61]. For underweight children, an explanation may be that parents encourage or do not restrict them from eating more sweets in order to gain weight [62]. It has also been suggested that increased satiety after eating sweetened foods may lead to reduced consumption of other foods, resulting in a lower weight or BMI [63]. As the available research shows, focusing solely on the frequency of consumption of sweetened foods is reasonable from a dental health perspective, but may be misleading in dietary counseling and health promotion. A whole diet approach should be considered, taking into account the portion sizes of these foods [48].

Most of the children studied showed adherence to the MediumDF pattern, and they were mainly boys. Girls predominate in the LowDF, HighDF, and HighSweets patterns, while, for example, among Australian adolescents, it was boys who consumed more energy from discretionary products than girls [64]. Boys have a significantly higher preference for fatty and sugary foods [64] but this has not been confirmed among Polish adolescents, where boys showed more moderation.

It is believed that one of the important determinants of dietary habits is the place of residence. In this research, children from mid-sized cities were less likely to belong to the LowDF pattern, but more likely to belong to the MediumDF pattern than children from rural areas. Some authors have found that the size of the agglomeration (urban–rural) is primarily important in determining one’s diet [65,66], rather than in differentiating DF consumption, especially sweets and salty snacks [67,68,69,70,71,72]. In our study, the differences were noticeable.

The differences in practices between cities and villages may be due to the specifics of retail practices [73]. The placement of discretionary foods such as soft drinks, chips, chocolate, and candy at the end of the aisle and near the checkout was equally common in each of the urban, suburban, and rural/non-metropolitan settings studied. However, urban stores had an overall healthier food environment compared to suburban and rural/non-metropolitan stores. Urban stores had more shelf space for fruits and vegetables and a lower percentage of soft drinks and candy at checkout. Cameron [73] confirmed that socio-economic differentiation is also present in the store environment of urban, suburban, and rural/non-metropolitan stores. This is also evident in Poland due to the rules of international retail chains and can affect discretionary food consumption.

Some authors emphasized that region and geographic location are more responsible for differences in diet than the level of urbanization [74,75]. Indeed, our research results showed the importance of regionalization. Children from the western macro-regions had significantly less adherence to LowDF, and those from the eastern macro-regions had greater adherence to MediumDF, but less adherence to HighSweets than those from the central region. A lifestyle study conducted in the southeastern region of Poland found that more than 30% of women and more than 40% of men were overweight, influenced mainly by economic status, but also by low physical activity and irregular meals, often explained by irregular work schedules and multiple responsibilities [76]. These studies included adults, but given the influence of the home situation on children’s behavior, it can be assumed that children follow the example of their caregivers in terms of lifestyle practices, especially nutrition [77,78]. However, these habits are not at risk in the presence of a MediumDF pattern. Moreover, there are no current data on the exact regionalization of DF consumption in Poland.

The analyzed lifestyle elements can be considered in the context of individual patterns because they are clearly related. Children in the LowDF and MediumDF patterns tended to be more physically active in their leisure time. The reasons for this may be attributed to attention to improving lifestyle and nutrition, given the higher likelihood of obesity among children in these patterns in the LowDF pattern. This is coordinated with low adherence to longer screen times and higher adherence to longer sleep and good nutritional knowledge. It is difficult to determine the exact reasons for these correlations, but they should be considered beneficial in terms of improving children’s lifestyles. What is needed, however, is a thorough analysis of the motives behind children’s behavior and the role of parents and schools in making these relationships work. The opposite relationship was observed in the HighDF pattern, which may be a kind of “disregard” for lifestyle factors and low attachment to overweight and obesity in this pattern. Underestimating the importance of physical activity and sleep may have health consequences in adulthood that children are, unfortunately, unaware of. Children in the HighSweets pattern had low adherence to high physical activity, but high adherence to moderate and good nutritional knowledge. They were also less likely to be overweight, which may be an important barrier to adopting a healthier lifestyle. The Slovenian study [78] confirmed this phenomenon and showed that although children participate in a variety of physical activities, including extracurricular activities and organized exercise programs in elementary schools, their level of physical activity is too low. Children spend a lot of time engaging in sedentary activities such as watching television, playing video games, and using the Internet. This is influenced by age, gender, socioeconomic status and parental support, school rules and environment, and access to safe outdoor areas for play and exercise [78]. A study on sedentary behavior, physical activity, and discretionary food consumption showed that children from highly educated mothers or high-income households were more likely to be allocated to the “relatively healthy lifestyle” cluster, while children with mothers with low-education or from low-income households were more likely to be allocated in the “high screen time and physically inactive” cluster [79]. A Spanish study reported that children spending at least one hour on daily leisure screen time had a higher prevalence of high-frequency discretionary food consumption than children exposed for less than one hour [80]. Low physical activity among Polish adolescents (regardless of screen time) was associated with higher odds of being overweight and central obesity, but not with muscle strength [81]. Some studies do not fully confirm such relationships [41,42]. Collaboration is therefore needed among all stakeholders involved in children’s education to raise awareness, promote positive behavior patterns, and identify risky behaviors. Further research is needed to develop effective strategies to prevent chronic diseases and enable children to lead healthier lifestyles. Analyses show that interventions aimed at personalized dietary counseling can reduce the proportion of discretionary foods and beverages in total energy, fat, sugar, and salt consumption [82].

### Strength and Limitations

The strength of this study is the use of a large, nationally representative sample of children aged 10–12 years. In addition, these are the most recent data collected in recent years (2022–2023) and may accurately reflect current patterns of DF consumption characteristic of this age group in Poland. Previously, studies have only looked at the habitual consumption of individual discretionary products in addition to those that are habitually consumed, whereas our study looks at the issue more broadly, taking into account a group of different factors. Furthermore, anthropometric measurements were objectively obtained by well-trained researchers using the same body composition analyzer, eliminating the possibility of reporting bias. In addition, this current study measured weight, height, and body composition (FM, MM, and FFM) to provide a broader picture when examining associations.

On the other hand, the cross-sectional nature of the study did not allow the causal relationship between the independent and outcome variables to be established; a longitudinal study would have addressed these shortcomings. Therefore, future studies should consider conducting longitudinal studies that can follow the same subjects over time to establish more robust causal relationships between the factors studied, discretionary food consumption, and obesity-related outcomes. Another limitation of this study is that it is not possible to assess why there is an increased consumption of discretionary foods and what factors besides socio-economic ones are involved. Furthermore, we did not investigate psychosocial determinants (e.g., stress, emotional eating, and parental feeding styles) in context to the reasons for discretionary food consumption. Hence, future research should focus not only on the factors determining discretionary food consumption in the context of excess body weight but also on psychosocial determinants that may explain this relationship.

## 5. Conclusions

Polish adolescents consume discretionary food, and its conditions and effects do not differ from the corresponding group in other countries of the world. DF consumption is related to body composition, socio-demographic factors, and lifestyle.

Given the global emphasis on the rationalization of daily diets, it seems necessary to implement intervention programs in Poland that would, among other things, clarify recommendations for the consumption of discretionary foods, following the example of other countries that have already achieved results in this regard. From a public health perspective, interventions to increase nutritional knowledge and improve lifestyles should be implemented with both adolescents and their parents in coordination with the school. The results of the study may help to identify the main predictors of DF consumption and may support the promotion of proper physical and social development of the young population in terms of developing appropriate dietary and lifestyle habits.

## Figures and Tables

**Table 1:** General characteristics of adolescents included in this study.

Characteristics	Girls		Boys		Total Group		*p*-Value *
Total	N	%	N	%	N	%	
	1469	100	1380	100	2849	100	
Age group (years)							
10	660	44.9	529	38.3	1189	41.7	0.0061
11	469	31.9	479	34.7	948	33.3	
12	340	23.2	372	27.0	712	25.0	
Place of residence:							
village	408	27.7	397	28.8	805	28.3	0.1119
≤100,000 residents	778	53.0	712	51.6	1490	52.3	
>100,000 residents	283	19.3	271	19.6	554	19.4	
Macroregions grouped:							
central	356	24.2	305	22.1	661	23.2	0.6389
eastern	705	48.0	677	49.1	1382	48.5	
western	408	27.8	398	28.8	806	28.3	
Leisure-time physical activity:							
low	149	10.1	184	13.3	333	11.7	<0.0001
moderate	715	48.7	494	35.8	1209	42.4	
high	605	41.2	702	50.9	1307	45.9	
Screen time [h/d]:							
<2	547	37.2	410	29.7	957	33.6	<0.0001
2–4	532	36.2	541	39.2	1073	37.7	
4–6	249	17.0	257	18.6	506	17.8	
>6	141	9.6	172	12.5	313	10.9	
Sleep time on an average weekday [h/d]:							
<6	145	9.9	121	8.8	266	9.3	0.0105
6–8	775	52.7	667	48.3	1442	50.6	
>8	549	37.4	592	42.9	1141	40.1	
Body mass status:							
underweight	119	8.1	148	10.7	267	9.4	<0.0001
normal weight	1074	73.1	874	63.3	1948	68.4	
overweight	165	11.2	200	14.5	365	12.8	
obesity	111	7.6	158	11.5	269	9.4	
Nutrition knowledge:							
low	384	26.1	414	30.0	798	28.0	0.0314
medium	996	67.8	871	63.1	1867	65.5	
high	89	6.1	95	6.9	184	6.5	

* Chi-Square test of independence.

**Table 2:** Characteristics of the patterns identified by frequency of consumption of discretionary foods; the mean ratings of the patterns on the classification variables.

Food Group	Total Group	Dietary Patterns				*p*-Value *
		LowDF N = 872	MediumDF N = 1003	HighDF N = 566	HighSweets N = 408	
Fast food	2.33 ± 1.11	1.74 ± 0.67 ^d^	2.30 ± 0.83 ^b^	3.48 ± 1.42 ^a^	2.09 ± 0.73 ^c^	<0.0001
Sugar-sweetened beverages	3.12 ± 1.56	1.67 ± 0.57 ^d^	3.49 ± 0.88 ^b^	5.18 ± 1.25 ^a^	2.42 ± 1.05 ^c^	<0.0001
Sweets or confectionery	3.89 ± 1.39	2.84 ± 0.96 ^d^	3.48 ± 0.76 ^c^	5.02 ± 1.25 ^b^	5.61 ± 0.70 ^a^	<0.0001
Salty snacks	3.13 ± 1.27	2.18 ± 0.76 ^c^	3.15 ± 0.89 ^b^	4.48 ± 1.26 ^a^	3.18 ± 1.17 ^b^	<0.0001

* *p*-values were calculated using ANOVA analysis of variance with the post-hoc Waller–Duncan k-ratio *t*-test; frequency scale: 1—less than once a month or never; 2—one-three times a month; 3—once a week; 4—three-four times a week; 5—once a day; and 6—a few times a day. ^a–d^ Superscript letters indicate statistically significant differences within rows.

**Table 3:** Characteristics of respondents by dietary patterns (N, %).

Variables		Total Group (N)	Dietary Patterns				*p*-Value *
			LowDF N = 872	MediumDF N = 1003	HighDF N = 566	HighSweets N = 408	
			%	%	%	%	
Gender							<0.0001
	Girls	1469	33.6	32.5	18.4	15.5	
	Boys	1380	27.5	38.0	21.5	13.0	
Age							0.0067
	10	1189	32.0	31.8	19.2	17.0	
	11	948	29.5	36.7	20.4	13.4	
	12	712	29.6	38.9	20.4	11.1	
Place of residence							0.0042
	village	766	33.9	32.6	19.0	14.5	
	≤100,000 residents	1429	27.8	37.4	21.8	13.0	
	>100,000 residents	654	33.4	33.2	15.9	17.5	
Macroregions grouped							0.0119
	central	1382	33.5	31.9	18.3	16.3	
	eastern	661	31.5	36.5	19.2	12.8	
	western	806	26.8	35.7	22.2	15.3	
Leisure-time physical activity							<0.0001
	low	333	25.5	25.3	26.4	22.8	
	medium	1209	38.8	18.9	27.4	14.9	
	high	1307	45.5	16.1	19.3	19.1	
Screen time [h/d]							<0.0001
	<2	957	47.7	9.8	24.6	17.9	
	2–4	1073	42.2	16.5	23.6	17.7	
	4–6	506	32.2	27.3	25.9	14.6	
	>6	313	24.3	35. 8	17.2	22.7	
Sleeping time on an average weekday [h/d]							<0.0001
	<6	266	32.0	26.3	23.3	18.4	
	6–8	1442	37.9	20.2	25.3	16.6	
	>8	1141	45.4	13.9	21.6	19.1	
Nutrition knowledge							<0.0001
	low	798	28.5	33.3	26.2	12.0	
	medium	1867	30.9	36.2	17.9	15.0	
	high	184	37.5	33.2	11.9	17.4	

* Chi-Square test of independence.

**Table 4:** Anthropometrics of the studied adolescents by patterns.

**Variables**	**Total Group** **N (%)**	**Dietary Patterns**				*p*-Value *
		**LowDF** **N = 872 (%)**	**MediumDF** **N = 1003 (%)**	**HighDF** **N = 566 (%)**	**HighSweets** **N = 408 (%)**	
BMI categories						
underweight	267 (100)	64 (25.8)	84 (31.5)	60 (22.5)	54 (20.2)	<0.0001
normal weight	1948 (100)	581 (29.8)	671 (34.5)	407 (20.9)	289 (14.8)	
overweight	365 (100)	119 (32.6)	139 (38.1)	62 (17.0)	45 (12.3)	
obese	269 (100)	103 (38.3)	109 (40.5)	37 (13.8)	20 (7.4)	
BMI (kg/m^2^)	18.82 ± 3.69	19.13 ± 3.88 ^a^	19.13 ± 3.82 ^a^	18.35 ± 3.29 ^b^	18.01 ± 3.23	<0.0001
Fat mass (FM) (%)	23.26 ± 6.42	24.01 ± 6.70 ^a^	23.47 ± 6.55 ^a^	22.35± 6.06 ^b^	22.43 ± 5.69 ^b^	<0.0001
Muscle mass (MM) (kg)	30.71 ± 6.41	30.96 ± 6.62 ^a^	31.40 ± 6.55 ^a^	30.07 ± 6.12 ^b^	29.31 ± 5.71 ^c^	<0.0001
Fat-free mass (FFM) (kg)	32.40 ± 6.74	32.67 ± 6.95 ^a^	33.13 ± 6.88 ^a^	31.74 ± 6.43 ^b^	30.95 ± 6.00 ^c^	<0.0001

* *p*-values were calculated using ANOVA analysis of variance with the post-hoc Waller–Duncan k-ratio *t*-test and the chi-squared test for categorical. ^a–c^ Superscript letters indicate statistically significant differences within rows.

**Table 5:** Associations between dietary patterns and selected characteristics of the study adolescents (odds ratios).

Variables	LowDF	MediumDF	HighDF	HighSweets
	OR (95% CI); *p*	OR (95% CI); *p*	OR (95% CI); *p*	OR (95% CI); *p*
Age [years]				
10	1	1	1	1
11	0.889 (0.74; 0.07); 0.2128	0.244 (1.04; 1.49); 0.0172	0.077 (0.87; 1.34); 0.4946	0.756 (0.59; 0.96); 0.0225
12	0.893 (0.73; 1.09) 0.2724	1.366 (1.13; 1.66) 0.0016	1.078 (0.85; 1.36) 0.5274	0.61 (0.46; 0.81) 0.0005
Place of residence				
villages	1	1	1	1
≤100,000 residents	0.75 (0.63; 0.9); 0.0023	1.237 (1.03; 1.48); 0.0211	1.189 (0.96; 1.47); 0.1144	0.88 (0.69; 1.13) 0.3121
>100,000 residents	0.977 (0.78; 1.23) 1.228	1.031 (0.82; 1.30) 0.7971	0.80 5(0.60; 1.07) 0.1395	1.248 (0.93; 1.68) 0.1392
Macroregions grouped				
central	1	1	1	1
eastern	0.915 (0.75; 1.11) 0.3752	1.224 (1.01; 1.49) 0.0439	1.064 (0.84; 1.35) 0.6113	0.752 (0.58; 0.98) 0.0315
western	0.729 (0.58; 0.91) 0.0058	1.186 (0.95; 1.47) 0.1255	1.274 (1.03; 1.65) 0.0655	0.922 (0.70; 1.22) 0.5726
Leisure-time physical activity				
low	1	1	1	1
medium	1.552 (1.16; 2.07) 0.0027	1.295 (1.04; 1.69) 0.0337	0.568 (0.43; 0.75) <0.0001	0.765 (0.56; 1.05) 0.1003
high	1.758 (1.32; 2.34) <0.0001	1.354 (1.04; 1.76) 0.0227	0.495 (0.38; 0.65) <0.0001	0.666 (0.48; 0.92) 0.0132
Screen time [h/d]				
<2	1	1	1	1
2–4	0.515 (0.43; 0.62) <0.0001	1.621 (1.35; 1.95) <0.0001	1.274 (1.03; 1.65) 0.0456	1.116 (0.87; 1.43) 0.3829
4–6	0.335 (0.26; 0.43) <0.0001	1.432 (1.43; 1.80) 0.0019	2.718 (2.06; 3.58) <0.0001	1.002 (0.74; 1.37) 0.9877
>6	0.182 (0.12; 0.46) <0.0001	0.811 (0.61; 1.08) 0.1548	7.183 (5.35; 9.65) <0.0001	0.849 (0.58; 1.25) 0.4040
Sleeping time on an average weekday [h/d]				
<6	1	1	1	1
6–8	1.383 (1.03; 1.87) 0.0338	1.19 (0.90; 1.57) 0.2218	0.575 (0.43; 0.77) 0.0002	0.975 (0.67; 1.43) 0.8987
>8	1.363 (1.01; 1.85) 0.0467	1.154 (0.87; 1.53) 0.3248	0.518 (0.38; 0.70) <0.0001	1.205 (0.82; 1.77) 0.3440
BMI categories				
normal weight	1	1	1	1
underweight	0.82 (0.61; 1.10) 0.1807	0.874 (0.66; 1.15) 0.3348	1.097 (0.81; 1.49) 0.5532	1.455 (1.05; 2.01) 0.0231
overweight	1.138 (0.90; 1.45) 0.2894	1.171 (0.93; 1.48) 0.1816	0.775 (0.58; 1.04) 0.0891	0.807 (0.58; 1.13) 0.2118
obese	1.46 (1.21; 1.90) 0.0050	1.297 (1.02; 1.68) 0.0492	0.604 (0.42; 0.87) 0.0066	0.461 (0.29; 0.74) 0.0013
Nutrition knowledge				
low	1	1	1	1
medium	1.122 (0.93; 1.35) 0.2152	1.135 (1.06; 3.34) 0.1552	0.616 (0.51; 0.75) <0.0001	1.29 (1.01; 1.65) 0.0443
high	1.509 (1.08; 2.11) 0.0163	0.992 (0.26; 2.32) 0.9625	0.383 (0.24; 0.61) <0.0001	1.54 (1.04; 2.38) 0.0428

## Data Availability

Due to ethical restrictions and participant confidentiality, the data cannot be released to the public. However, the survey data are available upon request to researchers who meet the criteria for access to confidential data. Requests for data access can be addressed to the coordinator of the Junior-Edu-Żywienie (JEŻ) project (Krystyna Gutkowska).

## References

[1] Australian Dietary Guidelines (2013). Australian Dietary Guidelines.

[2] Coxon C., Devenish G., Ha D., Do L., Scott J.A. (2020). Sources and Determinants of Discretionary Food Intake in a Cohort of Australian Children Aged 12–14 Months. Int. J. Environ. Res. Public Health.

[3] Johnson B.J., Bell L.K., Zarnowiecki D., Rangan A.M., Golley R.K. (2017). Contribution of Discretionary Foods and Drinks to Australian Children’s Intake of Energy, Saturated Fat, Added Sugars and Salt. Children.

[4] Sluik D., van Lee L., Engelen A.I., Feskens E.J. (2016). Total, Free, and Added Sugar Consumption and Adherence to Guidelines: The Dutch National Food Consumption Survey 2007–2010. Nutrients.

[5] Matthiessen J., Fagt S. Kostens Betydning for Børn og Unges Sundhed og Overvægt: 2000–2013. E-Artik. Fra DTU Fødevareinstitutet 2017. https://www.food.dtu.dk/english/-/media/institutter/foedevareinstituttet/publikationer/pub-2013/e-artikel_kostens-betydning-for-boerns-sundhed-og-overvaegt.pdf.

[6] Bell A.C., Kremer P.J., Magarey A.M., Swinburn B.A. (2005). Contribution of ‘noncore’ foods and beverages to the energy intake and weight status of Australian children. Eur. J. Clin. Nutr..

[7] Strazzullo P., Campanozzi A., Avallone S. (2012). Does salt intake in the first two years of life a_ect the development of cardiovascular disorders in adulthood?. Nutr. Metab. Cardiovasc. Dis..

[8] Kaikkonen J.E., Mikkilä V., Magnussen C.G., Juonala M., Viikari J.S., Raitakari O.T. (2013). Does childhood nutrition influence adult cardiovascular disease risk?—Insights from the Young Finns Study. Ann. Med..

[9] World Health Organization (2015). Guideline: Sugars Intake for Adults and Children.

[10] Pereira-da-Silva L., Rêgo C., Pietrobelli A. (2016). The Diet of Preschool Children in the Mediterranean Countries of the European Union: A Systematic Review. Int. J. Environ. Res. Public Health.

[11] Malik V.S., Hu F.B. (2019). Sugar-Sweetened Beverages and Cardiometabolic Health: An Update of the Evidence. Nutrients.

[12] Rauber F., Louzada M.L.D.C., Martinez Steele E., Rezende L.F.M., Millett C., Monteiro C.A., Levy R.B. (2019). Ultra-processed foods and excessive free sugar intake in the UK: A nationally representative cross-sectional study. BMJ Open.

[13] Keast D.R., Fulgoni V.L., Nicklas T.A., O’Neil C.E. (2013). Food Sources of Energy and Nutrients among Children in the United States: National Health and Nutrition Examination Survey 2003–2006. Nutrients.

[14] Grieger J.A., Wycherley T.P., Johnson B.J., Golley R.K. (2016). Discrete strategies to reduce intake of discretionary food choices: A scoping review. Int. J. Behav. Nutr. Phys. Act..

[15] Sutherland R., Nathan N., Brown A., Yoong S., Reynolds R., Walton A., Janssen L., Desmet C., Lecathelinais C., Gillham K. (2020). A cross-sectional study to determine the energy density and nutritional quality of primary-school children’s lunchboxes. Public. Health Nutr..

[16] Snetselaar L.G., de Jesus J.M., DeSilva D.M., Stoody E.E. (2021). Dietary Guidelines for Americans, 2020–2025: Understanding the Scientific Process, Guidelines, and Key Recommendations. Nutr. Today.

[17] Briefing Paper on Discretionary Foods Food Standards Scotland (2018). Food Standards Scotland Nutrition Science and Policy Branch. https://www.foodstandards.gov.scot/downloads/FSS_-_Discretionary_Foods_Paper_-_September_2018_final_for_publication.pdf.

[18] Cramer-Nielsen A., Bestle S.M.S., Biltoft-Jensen A.P., Matthiessen J., Lassen A.D., Christensen B.J., Gibbons S.J., Trolle E. (2022). Comparison of Discretionary Food and Drink Intake Based on a Short Web-Based Sugar-Rich Food Screener and a Validated Web-Based 7-Day Dietary Record. Nutrients.

[19] Biltoft-Jensen A., Matthiessen J., Hess Ygil K., Christensen T. (2022). Defining Energy-Dense, Nutrient-Poor Food and Drinks and Estimating the Amount of Discretionary Energy. Nutrients.

[20] The Danish Official Dietary Guidelines 2012. Ministry of Food, Agriculture and Fisheries of Denmark. https://en.fvm.dk/news-and-contact/focus-on/the-danish-official-dietary-guidelines.

[21] James-Martin G., Baird D.L., Hendrie G.A. (2021). Strategies to Reduce Consumption of Unhealthy Foods and Beverages: Scenario Modeling to Estimate the Impact on the Australian Population’s Energy and Nutrient Intakes. J. Acad. Nutr. Diet..

[22] Mauch C.E., Golley R.K., Hendrie G.A. (2024). Variety Predicts Discretionary Food and Beverage Intake of Australian Adults: A Cross-Sectional Analysis of an Online Food Intake Survey. J. Acad. Nutr. Diet..

[23] Hadjikakou M. (2017). Trimming the excess: Environmental impacts of discretionary food consumption in Australia. Ecol. Econ..

[24] (2023). Nordic Nutrition Recommendations. Integrating Environmental Aspects. Nordic Council of Ministers. https://pub.norden.org/nord2023-003/.

[25] Maneschy I., Jimeno-Martínez A., Miguel-Berges M.L., Rupérez A.I., Ortega-Ramiréz A.D., Masip G., Moreno L.A. (2024). Eating Behaviours and Dietary Intake in Children and Adolescents: A Systematic Review. Curr. Nutr. Rep..

[26] Blyth F., Haycraft E., Peral-Suarez A., Pearson N. (2025). Tracking and changes in the clustering of physical activity, sedentary behavior, diet, and sleep across childhood and adolescence: A systematic review. Obes. Rev..

[27] Fletcher E.A., McNaughton S.A., Crawford D., Cleland V., Della Gatta J., Hatt J., Dollman J., Timperio A. (2018). Associations between sedentary behaviours and dietary intakes among adolescents. Public. Health Nutr..

[28] Dhammawati F., Fagerberg P., Diou C., Mavrouli I., Koukoula E., Lekka E., Stefanopoulos L., Maglaveras N., Heimeier R., Karavidopoulou Y. (2023). Ultra-Processed Food vs. Fruit and Vegetable Consumption before and during the COVID-19 Pandemic among Greek and Swedish Students. Nutrients.

[29] Trigueros R., Aguilar-Parra J.M. (2025). Dietary and Sedentary Behavior in Children and Adolescents. Nutrients.

[30] Guerriero M.A., Dipace A., Monda A., De Maria A., Polito R., Messina G., Monda M., di Padova M., Basta A., Ruberto M. (2025). Relationship Between Sedentary Lifestyle, Physical Activity and Stress in University Students and Their Life Habits: A Scoping Review with PRISMA Checklist (PRISMA-ScR). Brain Sci..

[31] Hamulka J., Czarniecka-Skubina E., Gutkowska K., Drywień M.E., Jeruszka-Bielak M. (2024). Nutrition-Related Knowledge, Diet Quality, Lifestyle, and Body Composition of 7–12-Years-Old Polish Students: Study Protocol of National Educational Project Junior-Edu-Żywienie (JEŻ). Nutrients.

[32] Hamułka J., Wadolowska L., Hoffmann M., Kowalkowska J., Gutkowska K. (2018). Effect of an Education Program on Nutrition Knowledge, Attitudes toward Nutrition, Diet Quality, Lifestyle, and Body Composition in Polish Teenagers. The ABC of HealthyEating Project: Design, Protocol, and Methodology. Nutrients.

[33] Kowalkowska J., Wadolowska L., Hamulka J., Wojtas N., Czlapka-Matyasik M., Kozirok W., Bronkowska M., Sadowska J., Naliwajko S., Dziaduch I. (2019). Reproducibility of a Short-Form, Multicomponent Dietary Questionnaire to Assess Food Frequency Consumption, Nutrition Knowledge, and Lifestyle (SF-FFQ4PolishChildren) in Polish Children and Adolescents. Nutrients.

[34] Pyramid of Healthy Nutrition and Lifestyle for Children and Adolescents (4–18 Years). https://ncez.pzh.gov.pl/dzieci-i-mlodziez/podstawowe-zasady-zdrowego-zywienia/.

[35] Kułaga Z., Różdżyńska-Świątkowska A., Grajda A., Gurzkowska B., Wojtyło M., Góźdź M., Świąder-Leśniak A., Litwin M. (2015). Percentile charts for growth and nutritional status assessment in Polish children and adolescents from birth to 18 year of age. Stand. Med. Pediat..

[36] Tanita Manual. Tanita Manual: Children Body Fat. http://www.tanitapolska.pl/baza-wiedzy/analiza-wynikow-pomiarowych.

[37] Kromeyer-Hauschild K., Wabitsch M., Kunze D., Geller F., Geiß H., Hesse V., von Hippel A., Jaeger U., Johnsen D., Korte W. (2001). Perzentile für den Body-Mass-Index für das Kindes- und Jugendalter unter Heranziehung verschiedener deutscher Stichproben. Monatsschr. Kinderheilkd..

[38] Kim A., Kim J., Kye S. (2018). Sugar-sweetened beverage consumption and influencing factors in Korean adolescents: Based on the 2017 Korea youth risk behaviour web-based survey. J. Nutr. Health.

[39] Southerland J.L., Dula T.M., Slawson D.L. (2019). Barriers to healthy eating among high school youth in Rural Southern Appalachia. J. Appalach. Health.

[40] Beal T., Morris S., Tumilowicz A. (2019). Global patterns of adolescent fruit, vegetable, carbonated soft drink, and fast-food consumption: A meta-analysis of global school-based student health surveys. Food Nutr. Bull..

[41] Fayet-Moore F., McConnell A., Tuck K., Petocz P., Cassettari T., Rahimi-Ardabili H., Blumfield M., Marshall S. (2022). Patterns of discretionary food intake among Australian children and their association with socio-demographic, lifestyle, and adiposity measures. Nutr. Diet..

[42] Paulsen M.M., Myhre J.B., Totland T.H., Andersen L. (2023). Discretionary foods and drinks in Norwegian children and adolescents’ diet: Data from the national dietary survey Ungkost 3. Public Health Nutr..

[43] Ra J.S. (2022). Consumption of sugar-sweetened beverages and fast foods deteriorates adolescents’ mental health. Front Nutr..

[44] WHO (2023). Carbohydrate Intake for Adults and Children: WHO Guideline. https://www.who.int/publications/i/item/9789240073593.

[45] Leary N., Parker M., Rincón Gallardo Patiño S., Kraak V.I. (2024). An Evaluation of Healthy Hydration Recommendations for 93 Countries with Sugary Beverage Tax Legislation Globally, 2000–2023. Nutrients.

[46] Jodkowska M., Oblacinska A., Tabak I., Radiukiewicz K. (2011). Differences in dietary patterns between overweight and normal-weight adolescents. Dev. Period. Med.-Med. Wieku. Rozwoj..

[47] Gasser C., Mensah F.K., Russell M., Dunn S., Wake M. (2016). Confectionery consumption and overweight, obesity, and related outcomes in children and adolescents: A systematic review and meta-analysis. Am. J. Clin. Nutr..

[48] Lommi S., Figueiredo R.A.O., Tuorila H., Viljakainen H. (2020). Frequent use of selected sugary products associates with thinness, but not overweight during preadolescence: A cross sectional study. Brit. J. Nutr..

[49] Pesch M.H., Miller A., Appugliese D.P., Rosenblum K.L., Lumeng J.C. (2018). Mothers of Obese Children Use More Direct Imperatives to Restrict Eating. J. Nutr. Educ. Behav..

[50] Goossens L., Braet C., Van Vlierberghe L., Mels S. (2009). Loss of Control over Eating in Overweight Youngsters: The Role of Anxiety, Depression and Emotional Eating. Eur. Eat. Disord. Rev..

[51] Lutter M., Nestler E.J. (2009). Homeostatic and Hedonic Signals Interact in the Regulation of Food Intake. J. Nutr..

[52] Reichenberger J., Kuppens P., Liedlgruber M., Wilhelm F.H., Tiefengrabner M., Ginzinger S., Blechert J. (2018). No Haste, More Taste: An EMA Study of the Effects of Stress, Negative and Positive Emotions on Eating Behavior. Biol. Psychol..

[53] Devonport T.J., Nicholls W., Fullerton C. (2019). A Systematic Review of the Association between Emotions and Eating Behaviour in Normal and Overweight Adult Populations. J. Health Psychol..

[54] Zhou Y., Tse C.-S. (2020). The Taste of Emotion: Metaphoric Association between Taste Words and Emotion/Emotion-Laden Words. Front. Psychol..

[55] Zhou Y., Tse C.-S. (2022). Sweet Taste Brings Happiness, but Happiness Does Not Taste Sweet: The Unidirectionality of Taste-Emotion Metaphoric Association. J. Cogn. Psychol..

[56] Murray S.B., Griffiths S., Hazery L., Shen T., Wooldridge T., Mond J.M. (2016). Go Big or Go Home: A Thematic Content Analysis of Pro-Muscularity Websites. Body Image.

[57] Ganson K.T., Cunningham M.L., Pila E., Rodgers R.F., Murray S.B., Nagata J.M. (2022). Characterizing Cheat Meals among a National Sample of Canadian Adolescents and Young Adults. J. Eat. Disord..

[58] Kambanis P.E., Kuhnle M.C., Wons O.B., Jo J.H., Keshishian A.C., Hauser K., Becker K.R., Franko D.L., Misra M., Micali N. (2020). Prevalence and Correlates of Psychiatric Comorbidities in Children and Adolescents with Full and Subthreshold Avoidant/Restrictive Food Intake Disorder. Int. J. Eat. Disord..

[59] Rankin J., Matthews L., Cobley S., Han A., Sanders R., Wiltshire H.D., Baker J.S. (2016). Psychological Consequences of Childhood Obesity: Psychiatric Comorbidity and Prevention. Adolesc. Health Med. Ther..

[60] Kerem L., Van De Water A.L., Kuhnle M.C., Harshman S., Hauser K., Eddy K.T., Becker K.R., Misra M., Micali N., Thomas J.J. (2022). Neurobiology of Avoidant/Restrictive Food Intake Disorder in Youth with Overweight/Obesity Versus Healthy Weight. J. Clin. Child Adolesc. Psychol..

[61] Webber L., Hill C., Saxton J., Van Jaarsveld C., Wardle J. (2009). Eating behaviour and weight in children. Int. J. Obes..

[62] Huus K., Brekke H., Ludvigsson J.F., Ludvigsson J. (2009). Relationship of food frequencies as reported by parents to overweight and obesity at 5 years. Acta. Paediatr..

[63] Pei Z., Flexeder C., Fuertes E., Standl M., Buyken A., Berdel D., von Berg A., Lehmann I., Schaaf B., Heinrich J. (2014). Food intake and overweight in school-aged children in Germany: Results of the GINIplus and LISAplus studies. Ann. Nutr. Metab..

[64] Blumfield M., McConnell A., Petocz P., Rouf A., Duve E., Teasdale S., Marshall S., Fayet-Moore F. (2023). Relationship between discretionary food intake and sex, body image, health, and geographical remoteness among Indigenous Australian adolescents. Nutr. Diet..

[65] Rogalska-Niedźwiedź M., Charzewska J., Chabros E., Chwojnowska Z., Wajszczyk B., Zacharewicz E. (2008). Sposób żywienia dzieci czteroletnich ze wsi na tle dzieci z miast (Nutrition od 4-year old children from rural and urban environments). Probl. Hig. Epidemiol..

[66] Chęcińska Z., Krauss H., Hajduk M., Białecka-Grabarz K. (2013). Ocena sposobu żywienia młodzieży wielkomiejskiej i obszarów wiejskich (Assessment of eating habits in urban and rural youth). Probl. Hig. Epidemiol..

[67] Roma-Giannikou E., Adamidis D., Gianniou M., Nikolara R., Matsaniotis N. (1997). Nutritional survey in Greek children: Nutrient intake. Eur. J. Clin. Nutr..

[68] Tognarelli M., Picciolli P., Vezzosi S., Isola A., Moretti F., Tommassetto E., Fantuzzi A.L., Bedogni G. (2004). Nutritional status of 8-year-old rural and urban Italian children: A study in Pistoia, Tuscany. Int. J. Food. Sci. Nutr..

[69] Yannakoulia M., Karayiannis D., Terzidou M., Kokkevi A., Sidossis L.S. (2004). Nutrition-related habits of Greek adolescents. Eur. J. Clin. Nutr..

[70] Kelishadi R., Ardalan G., Gheiratmand R., Gouya M.M., Razaghi E.M., Delavari A., Majdzadeh R., Heshmat R., Motaghian M., Barekati H. (2007). Association of physical activity and dietary behaviours in relation to the body mass index in a national sample of Iranian children and adolescents: CASPIAN Study. Bull. World Health Organ..

[71] Hoffmann K., Bryl W., Marcinkowski J.T., Rzesoś A., Wojtyła E., Pupek-Musialik D. (2012). Dietary behaviours of adolescents from urban and rural areas in the district of Szamotuły-a preliminary study. Ann. Agric. Environ. Med..

[72] Ferreira A., Kęska A., Bogucka A. (2019). Comparison of selected lifestyle components in 12 year old children from Warsaw and Zamosc. Part II: Influence of physical activity, forms of spending leasure time, length of sleep for nutritional status of adolescents. Med. Og. Nauk. Zdr..

[73] Cameron A. (2018). The shelf space and strategic placement of healthy and discretionary foods in urban, urban-fringe and rural/non-metropolitan Australian supermarkets. Public Health Nutr..

[74] Oblacińska A., Tabak I., Jodkowska M. (2007). Demograficzne i regionalne uwarunkowania niedoboru masy ciała u polskich nastolatków. (Demographic and regional determinants of underweight in polish teenagers). Przegl. Epidemiol..

[75] Lazzeri G., Giacchi M., Spinelli A., Pammolli A., Dalmasso P., Nardone P., Lamberti A., Cavallo F. (2014). Overweight among students aged 11-15 years and its relationship with breakfast, area of residence and parents’ education: Results from the Italian HBSC 2010 cross-sectional study. Nutr. J..

[76] Domański M., Domańska A., Chęcińska-Maciejewska Z., Lachowicz-Wiśniewska S., Żukiewicz-Sobczak W., Weiner M. (2023). Health and nutritional behavior of a selected group of south-east Poland patients–A pilot study. Health Probl. Civiliz..

[77] Mahmood L., Flores-Barrantes P., Moreno L., Manios Y., Gonzalez-Gil E.M. (2021). The Influence of Parental Dietary Behaviors and Practices on Children’s Eating Habits. Nutrients.

[78] Lekše R., Godec D., Prosen M. (2023). Determining the Impact of Lifestyle on the Health of Primary School Children in Slovenia Through Mixed Membership Focus Groups. J. Community Health.

[79] Yang-Huang J., Van Grieken A., Wang L., Jansen W., Raat H. (2020). Clustering of Sedentary Behaviours, Physical Activity, and Energy-Dense Food Intake in Six-Year-Old Children: Associations with Family Socioeconomic Status. Nutrients.

[80] Avery A., Anderson C., McCullough F. (2017). Associations between children’s diet quality and watching television during meal or snack consumption: A systematic review. Matern. Child Nutr..

[81] Górnicka M., Hamulka J., Wadolowska L., Kowalkowska J., Kostyra E., Tomaszewska M., Czeczelewski J., Bronkowska M. (2020). Activity–Inactivity Patterns, Screen Time, and Physical Activity: The Association with Overweight, Central Obesity and Muscle Strength in Polish Teenagers. Report from the ABC of Healthy Eating Study. Int. J. Environ. Res. Public Health.

[82] Livingstone K.M., Celis-Morales C., Navas-Carretero S., San-Cristobal R., Forster H., Woolhead C., O’Donovan C., Moschonis G., Manios Y., Traczyk I. (2021). Personalised nutrition advice reduces intake of discretionary foods and beverages: Findings from the Food4Me randomised controlled trial. Int. J. Behav. Nutr. Phys. Act..

